# Direct Relationship between Interleukin-10 Gene Polymorphism and Hepatocellular Carcinoma Complicated by Direct Acting Antiviral Treatment of Hepatitis C Virus

**DOI:** 10.31557/APJCP.2021.22.10.3203

**Published:** 2021-10

**Authors:** Samar Ebrahim Ghanem, Nashwa Abuel-Fetuh Shebl, Ibrahim El Tantawy El Sayed, Hamed Mohamed Abdel Bary, Basant Farid Saad, Warda Othman Saad

**Affiliations:** 1 *Department of Clinical Biochemistry and Molecular Diagnostics, National Liver Institute, Menoufia University, Egypt. *; 2 *Hepatology and Gastoentrology Department, National Liver Institute, Menoufia University, Egypt. *; 3 *Chemistry Department, Faculty of Science, Menoufia University, Shebin El Koom, Egypt. *

**Keywords:** (IL-10) Interleukin-10, (HCC) Hepatocellular carcinoma, (HCV) Hepatitis C virus

## Abstract

**Objective:**

This study aimed to assess the correlation between the genotyping of interleukin-10 (IL-10 polymorphism rs 1800871) and the incidence hepatocellular carcinoma (HCC) among patients with hepatitis C virus (HCV) treated with direct acting antivirals (DAAs).

**Method:**

For 200 patients with HCV infection who completed DAA treatment and followed up for 1 year, IL-10 polymorphism SNP(-819) rs 1800871 analysis was conducted via real time polymerase chain reaction. During the follow-up period, 100 patients who developed HCC were selected and compared with 100 patients who did not develop any complications.

**Results:**

The studied patients were divided into two groups according to the incidence of complications after completion of DAA treatments. During the follow-up, 100 patients with HCV infection who developed HCC were selected and compared with 100 patients with HCV infection who did not develop any complications (positive control group). For the HCC group (n = 100), the mean age was 58.1 ± 6.4 years, with 92.7% being male and 7.3% being female; 91% had cirrhosis, 10% had lymphadenitis, 75% had splenomegaly, and 17% had ascites. In the positive control group (n = 100), mean age was 46.3 ± 9.4 years, with 68% being male and 32% being female; 20% had cirrhosis, 12% had splenomegaly, and 4.2% had ascites. The results demonstrated that sofosbuvir (SOF) + daclatasvir + ribavirin regimen was the most prevalent drug treatment for patients with HCC (72%), while SOF + Simeprevir was the most safe treatment for HCV infection among patients with HCC (2%). CT genotype was the most common genotype in the HCC group (56%), among different drug regimen (67.8%). T allele was the most prevalent in HCC group (61%), while the C allele was the least prevalent (39%). Conclusion: IL-10 genotyping may help in selecting the safest and most accurate drug regimen according to the safest genotype response relationship and follow-up of genotype resistance.

## Introduction

Worldwide, hepatocellular carcinoma (HCC) is the fifth most common cancer and the third most common cause of cancer-related deaths (Mckiver et al., 2020). The aim of this research was to identify the status of HCC in Egypt from different perspectives, in addition to the research strategy. Deep understanding of these aspects can help customize of hepatitis C virus (HCV) as the major explanation for HCC. The risk of developing HCC increases with the severity of liver inflammation and hepatic fibrosis. Reportedly, a balance between the release of pro- and anti-inflammatory cytokines determines the clinical course of HCV. Therefore, the risk of developing HCC cannot be ruled out. IL-10 and tumor necrosis factor alpha (TNF-α) play key roles within T-helper cell1 (Th1) and T-helper cell 2 balance during the inflammatory response against HCV (Rashed et al., 2020). HCC is a multifactorial disease, with several major risk factors contributing to liver carcinogenesis. Chronic inflammation caused by the activity of several inflammatory mediators has recently been identified as a cause of carcinogenesis (Dallio et al., 2021). Reportedly, gene polymorphisms in TNF-α and IL-10 are related to increased risk of HCC development in patients with chronic HCV infection. Certain variants are associated with more severe inflammation of the liver. This is mediated by Th1 cytokines and may increase the risk of developing HCC with an adverse prognosis (Aroucha et al., 2016). The emergence of DAAs, nonstructural protein 5A (NS5A), nonstructural protein 5B(NS5B) polymerase, and nonstructural protein3/4A (NS3/4A) protease inhibitors have paved a way for viral eradication rather than mass HCV treatment (Asselah et al., 2016). Despite the unparalleled success rates of DAAs, there is still an unmet need for more specific studies regarding relapses following treatment with these highly expensive drug regimens (Arias et al., 2017).

IL-10 is a crucial contra-inflammatory cytokine that induces the down regulation of pro-inflammatory cytokines (George et al., 2015). In contrast to many studies supporting the role of chemokines within the initiation and effector phases of inflammation, there is little evidence suggesting an anti-inflammatory role for chemokines (Poordad et al., 2016). It is difficult to assess the role of HCV eradication on the incidence and recurrence of HCC (Reig et al., 2016; Conti et al., 2016). The Barcelona Clinic Liver Cancer (BCLC) system is unique in that manner. It seeks to determine patient prognosis, as well as recommends specific treatment algorithms based on HCC tumor stage (Tsilimigras et al., 2019; Bruix et al., 2016; Saillard et al., 2020).

## Materials and Methods

This study enrolled 200 patients with HCV infection. These patients were enrolled if they completed their DAA treatment and followed up for 1 year after the end of treatment. Patients were recruited from Sovaldi clinic National Liver Institute (NLI), Menoufia University from April 2018 to August 2019. During the follow-up, 100 patients with HCV infection developed HCC. Another 100 patients with HCV who had no complications served as positive controls. According to the treatment regimen, 72 patients with HCC were treated with sofosbuvir (SOF) + daclatasvir (DAC) + ribavirin (RBV ), 15 with SOF+DAC, 11 with simeprevir (SIM)+SOF+ RBV, and 2 with SOF+SIM. Overall, 34 patients with HCV infection were treated with SOF+DAC, 44 with SIM+SOF+ RBV, and 22 with SOF + SIM. SOF was administered at a dose of 400 mg/day and DAC at a dose of 60 mg/day. SIM was administered at a dose of 150 mg once a day with food. RBV was supplied in 200-mg capsules, and the recommended dose was 1,200 mg daily if patient weight was >75 kg, and 1,000 mg daily if patient weight was <75 kg, given in two doses. RBV dose modification or discontinuation was allowed at the discretion of the treating physician according to change in hemoglobin level. Potential drug-drug interactions with patient’s medications were assessed using the University of Liverpool application on smart phones (Liverpool HEP iChart) or the website (http://www.hep- druginteractions.org/checker). This study was conducted in accordance with the International Conference on Harmonization guideline for good clinical practice and the ethical principles of the Declaration of Helsinki. The research ethics committee of the National Liver Institute, Menoufia University-Egypt approved the study, and written informed consent was obtained from all participants.

The following data were collected for all included cases: Laboratory investigations done for both groups Basic blood samples were collected before treatment and were analyzed for blood chemistry, hematology, and HCV ribonucleic acid (RNA) using the fully automated auto analyzer SYNCHRON CX9ALX (Randox, CA, USA), Sysmex K-21 (Sysmex Corporation, Kobe, Japan), and COBAS^®^ TaqMan^®^ HCV assay (Roche Molecular Diagnostics, CA, USA) with a lower limit of quantitation of <10 IU/mL, respectively. Follow-up blood samples were obtained from patients only for assessing transaminases. During treatment and at 12 months after treatment, a complete blood count and HCV RNA was conducted.

*Radiological data*

Liver cirrhosis, focal lesion, lymphadenitis, ascites, and splenomegaly were diagnosed based on ultrasound examination (for both groups) and computed tomography or a liver biopsy (for HCC group).

*Molecular analysis done for both groups*

For both groups, genomic DNA was extracted from the whole blood using a spin column method according to the manufacturer’s protocol (QIAamp Blood Kit, Qiagen).

*Genotyping by real time (PCR)*

IL-10 gene (-819) rs 1800871 was genotyped using the TaqMan allelic discrimination assay that detects variants of a single nucleic acid sequence (Applied Biosystems Real time fast7500, Thermo Fisher Scientific Inc., Life Technologies TM, CA, USA). The presence of two primer/probe pairs in each reaction allows for the genotyping of two possible variants at the SNP site in a target template sequence, using genotyping qPCR Master Mix (2×) and genotyping assay (primers and probe) 40 x (Thermo Fisher Scientific Inc.). Reaction master mix for amplification (total volume 20 µL) constituted 0.5 µL of genotyping assay, 10 µL of genotyping qPCR Master Mix, 3.5 µL of DNA se-free water, and 6 µL of genomic DNA template. For negative control reaction, 6 µL of DNA se-free water was added. 

The cycling parameters were set as follows: initial denaturation step at 94°C for 4 min, 35 cycles of denaturation at 94°C for 30 s, annealing and extension 60°C for 90 s.

*Statistical Analysis*

All data were statistically analyzed using the SPSS statistical software package version 22.0 (SPSS, Inc., Chicago, IL). Categorical variables were compared using the *x*^2^ test or Fisher’s exact test when appropriate. Two-group comparisons were performed using the Student’s t-test or Mann–Whitney U-test for parametrically or non-parametrically distributed data. For comparisons of more than two groups, analysis of variance was performed for parametrically or non-parametrically distributed data. Multivariate logistic regression using the stepwise backward approach was performed to identify the predictors of HCC incidence. The obtained results are presented as odds ratio (OR) with 95% confidence interval (CI). The variations were considered statistically significant when P was <0.05 (Zhang et al., 2016).

## Results

This study included 200 patients with chronic HCV infection. These patients completed their different DAA regimens. All patients were classified into two groups: HCC group (n = 100, patients with HCV infection who developed HCC during follow-up) and control group (n = 100; patients with HCV infection who completed their different DAA regimens without developing complications). All HCV patients were treated and followed up according to the standard clinical protocol for 1 year. Clinical findings and laboratory results of the studied groups are outlined below. Table 1 shows the correlation between the HCC group and HCV group with regard to age, laboratory findings, and clinical data for 100 patients with HCC. The mean age was 58.1 ± 6.4 years, and 91% of patients had cirrhosis, 10% had lymphadenitis, 75% had splenomegaly, and 17% had ascites. For the 100 patients with HCV infection, mean age was 46.3 ± 9.4 years, and 20% had cirrhosis, 12% had splenomegaly, and 4.2% had ascites.

**Figure 1:**
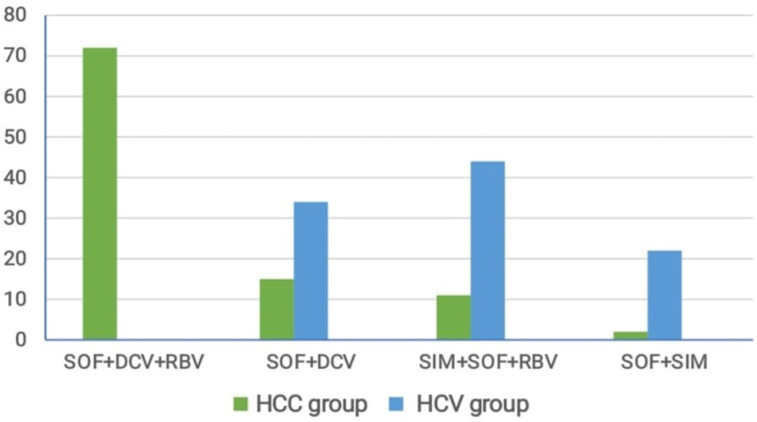
Comparison between Type of Drug Regimen in HCC and HCV Group

**Figure 2:**
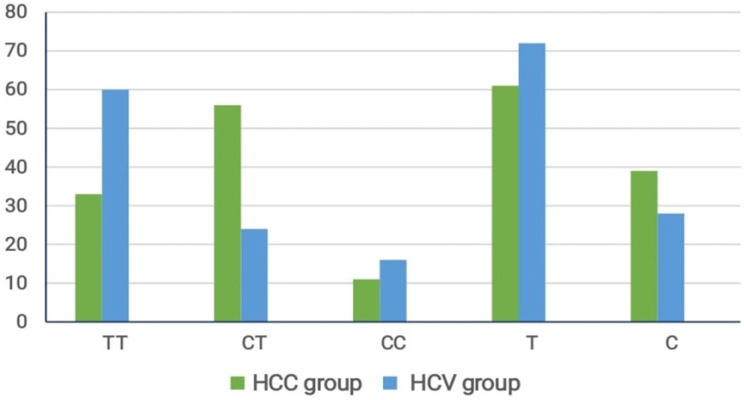
Correlation between Genotyping and Type of Drug Regimen in HCC Group

Figure 1 shows the distribution of different drug regimens in the HCC and HCV groups. Of all patients in HCC group, 72 were treated with SOF+DAC+RBV, 15 with SOF+DAC, 11 with SIM+SOF+RBV, and 2 with SOF+SIM. Of the patients with HCV infection, 34 were treated with SOF+ DAC, 44 with SIM+SOF+RBV, and 22 with SOF+SIM. These results explained that SOF + DAC +RBV regimen was the most prevalent drug treatment for HCC. Table 2 indicates the frequency of genotypes noted in studied groups. Overall, 33 patients with HCC had TT genotype, 56 had CT genotype, and 11 had CC genotype. Of the patients with HCV infection, 60 had TT genotype, 24 had CT genotype, and 16 had CC genotype. These results indicated that CT genotype is the most common genotype among patients with HCC. Figure 2 shows the correlation between different drug regimens and IL10 genotypes. Overall, 33 patients had TT genotype. Of these patients, 29 were treated with SOF+DAC+RBV, 3 with SOF+DAC, and 1 with SIM+SOF+RBV. In total, 56 patients had CT genotype; of these, 38 were treated with SOF+DAC+RBV, 10 with SOF+DAC, 6 with SIM+SOF+RBV, and 2 with SOF+SIM. Of 11 patients with CC genotype, 8 were treated with SOF+DAC+RBV, 2 with SOF+DAC, and 1 with SIM+SOF+RBV. These results indicate that the CT genotype is the commonest genotype among patients treated with different drug regimens.

Table 3 shows correlation between genotyping and TNM classification of malignant tumors. Child-Pugh classification, BCLC class, and HCC nodules in the HCC group in TNM classification, of 33 patients with TT genotype, 16 patients were categorized in class I, 12 in class II, 3 in class III, and 2 in class IV. Of 56 patients with CT genotype, 18 were categorized in class I, 32 in class II, 5 in class III, and 1 in class IV. Of 11 patients with CC genotype, 8 were categorized into class I, 3 in class II, 0 in class III, and 0 in class IV. Based on Child-Pugh classification, of 33 patients with TT genotype, 27 were categorized in class A, 6 in class B, and 0 in class C. Of 56 patients with CT genotype, 42 were categorized in class A, 11 in class B, and 3 in class C. Of 11 patients with CC genotype, 7 were categorized in class A, 3 in class B, and 1 in class C. Based on BCLC classification, of 33 patients had TT genotype, 11 were categorized in class 0, 4 in class A, 6 in class B, 12 in class C, and 0 in class D. Of 56 patients with CT genotype, 14 were categorized in class 0, 15 in class A, 4 in class B, 20 in class C, and 3 in class D. Of 11 patients with CC genotype, 1 patient was categorized in class 0, 3 in class A, 3 in class B, 3 in class C, and 1 in class D. Based on HCC nodules, of 33 patients with TT genotype, 28 had 1 nodule, 2 had 2-3 nodules, and 3 had >4 nodules. Of 56 patients with CT genotype, 45 had 1 nodule, 7 had 2-3 nodules, and 4 had >4 nodules. Of 11 patients with CC genotype, 10 had 1 nodule, 0 had 2-3 nodules, and 1 had >4 nodules.

Table 4 shows the results of multiple logistic regression analysis of HCC vs HCV groups. Age >60 years and male sex were the independent and significant predictive factors of increased risk of HCC (7.8 and 3.8 times, respectively). Allele C was found to be dominantly associated with increased risk of HCC development after adjusting for age and sex (OR [95% CI] = 2.3 [1.2–4.6], adjusted P value = 0.01). C/T genotype was associated with increased risk of HCC development after adjusting for age and sex (OR [95% CI] = 2.7[1.3 – 5.4], adjusted P value = 0.003).

**Table 1:** Correlation between HCC Group and HCV Group as Regards Age, Laboratory Investigation and Clinical Data

	HCC group N=100 No (%)	HCV group N=100 No (%)	U mann-whiteny test	P VALUE
Age (years)				
Mean± SD (min-max)	58.1±6.4	46.3±9.4	6.7	0.001
Range	45 – 75	30 – 62		
Sex:				
Male	89 (92.7%)	68 (68%)	15.2*	0.001
Female	7 (7.3%)	32 (32%)		
ALT (U/L)				
Mean ±SD (min – max)	44.6±27.8	34.4±13.5	1.95	0.05
Range	10 – 219	11 – 70		
AST (U/L)				
Mean ±SD (min – max)	51.3±33.4	29.8±10.3	5.6	0.001
Range	11 – 301	12 – 49		
Total bilirubin (mg/dL)			2.9	0.003
Mean ±SD (min – max)	1.3±0.98	1.01±0.69		
Range	0.2 – 3.0	0.4 – 2.9		
Direct bilirubin (mg/dL)			2.9	0.003
Mean ±SD (min – max)	0.43±0.63	0.46±0.42		
Range	0.1 – 3.0	1 – 1.8		
Albumin(gm/dL)			1.3	0.176
Mean ±SD (min – max)	3.5±0.86	3.6±0.94		
Range	1 – 5.1	1 – 4.9		
AFP (ng/dL)			8.3	0.001
Mean ±SD (min – max)	4332±16822	3±1.7		
Range	2 – 100000	1.5 – 6		
Pt concentration (S)			6.2	0.001
Mean ±SD (min – max)	79.2±14.7	93.6±9.2		
Range	40 – 106	60 – 100		
INR			0.483	0.624
Mean ±SD (min – max)	1.12±0.25	1.05±0.08		
Range	1 – 2.2	1 – 1.4		
CREAT. (mg/dL)			6.3	0.001
Mean ±SD (min – max)	1.01±0.22	0.81±0.22		
Range	0.5 – 2.0	0.5 – 1.5		
Relapsers	8 (8 %)	16 (16%)	0.601*	0.438
History of decompensation	21 (21%)	24 (24%)	0.248	0.594
Cirrhosis	91 (91%)	20 (20%)	78.8	0.001
Lymph nodes	10 (10%)	0 (0%)	5.3**	0.021
Splenomegaly	75 (75%)	12 (12%)	53.3	0.001
Ascites	17 (17%)	4 (4.2%)	4.8	0.029
HCC recurrence	3 (3%)	-	-	-

(HCC) hepatocellular carcinoma, (HCV) hepatitis C virus, (ALT) alanine aminotransferase, (AST) aspartate aminotransferase, (AFP) alpha fetoprotein, (Pt concentration) prothrombin concentration and (CREAT)Creatinine. *chi square test, U mann-whiteny test; **Fisher exact test; * Statistically significant at P < 0.05; Data are expressed as mean ± SD and median (min.—max.) for continuous variables.

**Table 2:** Correlation between Genotyping and Allele in HCC and HCV Group

	HCC group N=100 No (%)	HCV group N=100 No (%)	OR (95%CI)
Genotyping			4.2
TT*	33 (33%)	60 (60%)	(2.2 – 8.04)
CT	56 (56%)	24 (24%)	1.2
CC	11 (11%)	16 (16%)	(0.5 – 3.0)
Dominant			
TT	33 (33%)	60 (60%)	3.04
C/C + C/T	67 (67%)	40 (40%)	(1.7 – 5.4)
Recessive			
CC	11 (11%)	16 (16%)	0.6
C/T+T/T	89 (89%)	84 (84%)	(0.2 – 1.4)
Over-dominant			
CT	56 (56%)	24 (24%)	1.9
CC+T/T	44 (44%)	76 (76%)	(1.4 – 2.5)
Allele			
T	122 (61%)	144 (72%)	1.6
C	78 (39%)	56 (28%)	(1.1 – 2.5)

(HCC) hepatocellular carcinoma and (HCV) hepatitis C virus; * OR odds ratio, 95%CI (95% confidence interval ).

**Table 3:** Correlation between Genotyping and TNM, Child Classification, BCLC and HCC Nodules in HCC Group

	TT	CT	CC	P value
	(no=33)	(no=56)	(no=11)	
TNM				0.14
· I	16 48.5	18 32.1	8 72.7	
· II	12 36.4	32 57.1	3 27.3	
· III	3 9.1	5 8.9	0 0.0	
· IV	2 6.1	1 1.8	0 0.0	
Child-Pugh classification				0.555
·A	27 81.8	42 75.0	7 63.6	
· B	6 18.2	11 19.6	3 27.3	
· C	0 0.0	3 5.4	1 9.1	
BCLC				0.23
· O	11 33.3	14 25.0	1 9.1	
· A	4 12.1	15 26.7	3 27.2	
· B	6 18.1	4 7.1	3 27.2	
· C	12 36.4	20 35.7	3 27.2	
· D	0 0.0	3 5.3	1 9.1	
HCC nodules				0.675
· 1	28 84.8	45 80.4	10 90.9	
· 2-3	2 6.1	7 12.5	0 0.0	
· >4	3 91	4 7.1	1 9.1	

TNM, TNM Classification of Malignant Tumors; *BCLC:Barcelona-Clinic Liver Cancer; *HCC nodules:hepatocellular carcinoma nodules; * Statistically significant at P < 0.05

**Table 4:** Multiple Logistic Regression Analysis of Predictive Factors Using Dominant Genetic Model

	HCC and HCV	
	p-value	OR (95%CI)
Age (> 60 years)	0.001	7.8 (3.3 – 18.6)
Male Gender	0.005	3.8 (1.4 – 9.7)
dominant genetic model	0.01	2.3 (1.2 – 4.6)
T/T		
C/C+C/T		
Over dominant genetic model	0.003	2.7 (1.3 – 5.4)
C/T		
C/C+T/T		

*OR (odds ratio), 95%CI (95% confidence interval); * Statistically significant at P < 0.05

## Discussion

DAAs comprise a newer class of drugs used for the treatment of HCV (Arzumanyan et al., 2013). These drugs target HCV at a specific stage of life cycle. They have shorter treatment times, fewer side effects, and higher sustained virologic response (SVR) rates than other older drugs (Westbrook and Dusheiko, 2014). Most DAAs are administered in combination to improve their efficacy. In addition, RBV and peginterferon are sometimes added to DAAs to improve how it attacks the virus (Yang and Roberts, 2010). IL-10 is a cytokine with strong anti-inflammatory properties and plays an important role in limiting host immune response to diseases, thereby halting damage to the host and maintaining normal tissue balance (El-Serag, 2012). Fluctuations in IL-10 level led to enhanced immunopathology in response to infection as well as to increased risk of many autoimmune diseases (Heim et al., 2020). Thus, studying IL-10 gene expression and polymorphisms is crucial for understanding disease progression (Monteiro et al., 2020; D’Ambrosio and Colombo, 2016). The present study revealed highly significant differences between HCC and HCV groups regarding age, sex, creatinine levels, prothrombin time, alpha fetoprotein (AFP) level, aspartate amino aminotransferase (ALT) level (P value < 0.005), in agreement with a previous study (Aroucha et al., 2016) found highly statistically significant differences with AFP, AST, and total bilirubin levels (P value < 0.0001) between HCC and HCV groups. In regard to age, transferase (AST) level, splenomegaly, and bilirubin level (P value < 0.001 and < 0.003). In addition, the two groups differed significantly regarding alanine transaminase. In agreement with a previous study (Aroucha et al., 2016) that found a highly statistically significant difference between HCC and HCV groups with regard to age, AFP, AST, and total bilirubin levels (P value < 0.0001). There was no statistically significant difference between the groups regarding ALT level (P value = 0.788). In addition, a previous study (Saleh et al., 2020) revealed a highly significant difference between HCC and cirrhosis groups with regard to AFP, AST, (P value < 0.001, both), and ascites (P value < 0.006).On the contrary, they demonstrated no statistical significant difference between HCC and cirrhosis groups with regard to age (P value < 0.06), international normalized ratio (P value < 0.275), sex (P value < 0.102), lymphadenitis (P value < 0.089), splenomegaly (P value < 0.154), and bilirubin level (P value < 0.412). However, the groups differed significantly regarding albumin level (P value < 0.003).The present study observed a high statistically significant difference between HCC and HCV groups with regard to the type of drug regimen used; SOF+DAC+RBV was the most prevalent drug regimen in HCC group (P value < 0.001) and SOF+SIM was the safest drug regimen in the HCV group .These results are in agreement with a previous study of (Ghanem et al., 2020) who found that SOF+DAC+RBV was the most prevalent drug treatment for HCC. Similarly, another study (Abdelaziz et al., 2019) assured that SOF+DAC+RBV regimen was the most prevalent in HCC. In contrast, another study (Li et al., 2018) found that the SOF/SIM regimen was the most common with HCC. In contrast, Li et al., (2018) found SOF/SIM regimen was the most common one with HCC. The present study found a high statistically significant difference between HCC and HCV groups regarding genotyping, where CT and TT were the most common genotypes in HCC and HCV groups respectively, with CT being the most common genotype among different drug regimens (P value < 0.001). There was no statistically significant difference between the HCC and HCV groups regarding alleles (P value < 0.06). 

These results are in agreement with those of a previous study (Wang et al., 2019) who found that CT was the most common genotype in HCC group (P value < 0.05). Few studies (Aroucha et al., 2016; Wang et al., 2019) found that T allele was the most common allele in HCC. Moreover, reports (Li et al., 2018; Maurya et al., 2018) demonstrated that TT genotype of IL-10(−819) gene was found in 20 patients (50%) with HCV infection and 25 patients (62.5%) with HCC. In our study, we noted a high statistically significant difference between genotyping and type of drug regimen in the HCC group, where CT was the most common genotype among different drug regimens (P value < 0.001). Two studies (Saleh et al., 2020; Obada et al., 2017) found no statistically significant difference between various genotype patterns of IL-10 (-819 C/T) with regard to SVR in patients with HCV infection treated with PEG–IFN/RBV.In our study, there were no statistically significant differences between genotyping and TNM, Child-Pugh classification, BCLC class, and HCC nodules in the HCC group (P value < 0.140, 0.555, 0.23, and 0.675, respectively).Similarly, a previous study (Aroucha et al., 2016) found no statistically significant difference between genotyping and Child-Pugh classification, BCLC class, and HCC nodules in the HCC group (P value < 0.414, 0.861, 0.04, respectively).In contrast, a previous study (Saleh et al., 2020) found a high statistically significant difference between genotyping and BCLC classification in the HCC group (P value < 0.001). Contrary to our study, a previous study (Aroucha et al., 2016) found that TT genotype at −819 (rs1800871) of IL-10 was associated with the number of nodules and advanced stages of HCC according to the BCLC classification. Our study showed a high statistically significant difference between multiple logistic regression models of HCC and HCV regarding age and sex (P value < 0.001 and 0.005, respectively) and statistically significant difference regarding dominant genetic model (P value < 0.01).

These results were in accordance with those reported previously (Aroucha et al., 2016), where age and male sex increased the risk of HCC incidence (P value < 0.0001) and a statistically significant difference was noted with regard to the dominant genetic model (P value < 0.027). 

## Author Contribution Statement

Samar EbrahimGhanem, NashwaAbuel-Fetuh Shebl, Basant Farid Saad and Warda Othman Saad: Contributed to study concept, design, clinical investigations, methodology, data collection, statistical analysis and interpretation of the data, Ibrahim El Tantawy El Sayed and Hamed Mohamed Abdel Barry: Supervision and conceptualization in the study. All authors contributed to writing of the papers and critically revised and finalized paper and all authors read and approved the final manuscript.
